# Self-Reported Personality Functioning in Patients With Substance Use Disorders: Similarities and Specifics With Respect to the Czech Community Sample

**DOI:** 10.31083/AP46058

**Published:** 2025-08-26

**Authors:** Lucia Schlosserova, Livia Rosova, Tadeas S. Zbornik, Karel D. Riegel

**Affiliations:** ^1^Department of Addictology, 1st Faculty of Medicine, Charles University and General University Hospital in Prague, 128 00 Prague, Czech Republic; ^2^Department of Applied Mathematics and Statistics, Faculty of Mathematics, Physics and Informatics, Comenius University, 842 48 Bratislava, Slovakia

**Keywords:** personality, substance-related disorders, personality assessment, psychometrics

## Abstract

**Objective:**

Information about the level of general personality functioning could provide benefits for tailoring substance use disorder (SUD) treatment. This study examined self-reported personality functioning among patients with SUD compared to the general population, gender specifics, and the psychometric properties of the Czech Level of Personality Functioning Scale-Self Report (LPFS-SR).

**Methods:**

Two samples were used in this study. Sample 1 (n = 368) consisted of patients with SUD, while Sample 2 (n = 497) comprised volunteers from the general population. All participants, with an age range of 18–75 years, completed a battery of self-assessment tools, including a demographic form, the Personality Inventory for DSM-5 (PID-5), and the LPFS-SR, administered via a pencil-and-paper method. Internal consistency and several aspects of the validity of the Czech LPFS-SR were examined.

**Results:**

The LPFS-SR showed high internal consistency as estimated by Cronbach’s alpha (α ≥ 0.66) and the high mutual correlation with the PID-5 varied from 0.21 to 0.77. Principal component analysis (PCA) of the four LPFS-SR subscales indicated a single-component structure, accounting for 78.21% of the variance in Sample 1 and 79.20% in Sample 2, supporting previous results regarding the LPFS-SR factorial structure. Furthermore, gender-specific cut-off scores were obtained and are discussed in relation to previous research.

**Conclusion:**

The findings indicate that the Czech LPFS-SR is a valid and reliable tool with acceptable discriminating capacity. It can be used in research and clinical assessments of personality functioning in patients with SUD, particularly when considering gender-specific characteristics.

## Main Points

1. The Czech version of the Level of Personality Functioning Scale – Self Report (LPFS-SR) demonstrated high internal consistency and strong validity, making it a reliable tool for assessing personality functioning.

2. The LPFS-SR correlated strongly with the Personality Inventory for DSM-5 (PID-5), confirming its criterion validity and connection to established diagnostic frameworks.

3. A single-component structure was confirmed through Principal Component Analysis (PCA) in both clinical and non-clinical samples, explaining over 78% of the variance in both groups.

4. Gender-specific cut-off scores were established, providing clinically useful thresholds that may improve diagnostic accuracy and inform individualized treatment planning in substance use disorders (SUD) populations.

## 1. Introduction

Personality disorders (PDs) are among the most prevalent comorbidities linked to substance use disorders (SUDs) [1]. These findings underscore the importance of carefully considering therapeutic strategies and interventions tailored to this complex dual pathology. Nevertheless, the development of effective treatment strategies relies on the accurate diagnosis of both conditions, which can be particularly challenging in the case of PDs. As the level of personality pathology plays a key role in SUD treatment [2, 3], the ability to identify impairments in overall personality functioning that trigger dysfunctional behavioral patterns in individuals with SUD might have significant therapeutic importance [4]. People with SUD and severe personality disorders are associated with poorer treatment outcomes [5, 6] and more frequent relapses [7]. According to Brown* et al*. [8], one of the most consistent predictors of SUD treatment dropout and relapse is comorbidity of SUD and PD. The well-documented comorbidity of PDs and SUD (e.g., [9]) calls for a unified approach to personality pathology assessment with a focus on the degree of impairment in personality functioning.

Currently, the diagnosis of PDs is transitioning from a categorical to a dimensional approach, which places greater emphasis on overall personality functioning and its impairment rather than considering PDs as distinct, phenomenologically diagnostic entities. Research suggests that even if an individual no longer meets the criteria for a specific PD, functional impairment may persist [10]. In this regard, the fifth edition of the Diagnostic and Statistical Manual of Mental Disorders (DSM-5) has proposed a hybrid approach to PD diagnosis, the Alternative Model for Personality Disorders (AMPD), which defines personality pathology based on impairments in personality functioning (i.e., Criterion A). These impairments can be further specified by one or more pathological personality traits (i.e., Criterion B). Personality functioning refers to an individual’s capacity to navigate and manage personal and interpersonal challenges effectively. This includes sustaining a coherent self-concept, engaging in self-reflection, maintaining meaningful relationships with others, and interpreting and recognizing others’ emotions appropriately [11, 12]. However, in the context of the AMPD, personality functioning refers to a general impairment in relation to the self and others, and is closely related to psychological maturity. It indicates the extent to which an individual demonstrates psychological maturation through their ability to adapt effectively to their current environment [11, 13]. Personality functioning possesses several key properties that contribute to its significance in the study of personality pathology: (A) Universality refers to the understanding of personality functioning as a universal construct, which can be conceptualized as a single dimension [14]. (B) Core Component highlights its role as a central aspect of personality psychopathology [11], reflecting the degree to which an individual can relate to themselves and others. This capacity distinguishes between adaptive and maladaptive behavior. (C) Correlation with Personality Traits underscores the observed correlation between PD symptoms and maladaptive personality traits [14, 15]. (D) Overall Severity provides valuable information regarding the overall severity of personality problems. (E) Therapeutic Relevance emphasizes that personality functioning is generally thought to be somewhat less stable than personality traits [15]. This implies that therapeutic interventions should prioritize fostering psychological maturity rather than attempting to modify stable personality traits, enabling individuals to approach situations in a more adaptive and functional way.

The Level of Personality Functioning Scale- Self Report (LPFS-SR) directly corresponds with Criterion A of the AMPD and was developed as a self-assessment tool intended to complement a patient’s clinical profile, primarily based on the clinician’s observations and judgement [16]. The LPFS-SR examines disturbances in personality functioning both in relation to oneself (Identity, Self-direction) and others (Empathy, Intimacy). Identity evaluates an individual’s capacity to perceive themselves as distinct beings with stable self-esteem, a coherent self-concept, and the ability to self-evaluate and regulate emotions. Self-direction pertains to the ability to set and pursue life goals, uphold moral values, and engage in self-reflection. Empathy refers to an individual’s ability to understand the feelings and motivation of others, tolerate differing perspectives, and recognize their influence on others. Lastly, Intimacy examines the individual’s capacity to establish deep and long-term relationships [12]. Pathology or dysfunctions in these subdomains are present across all variants of PDs, depending on the severity of the disorder, and show high intercorrelation between each other [12, 17].

To ensure the efficacy of the LPFS-SR, psychometric studies have documented substantial correlation with other personality pathology assessment tools such as: General Assessment of Personality Disorder (GAPD); Personality Assessment Inventory (PAI); General Personality Pathology Scale (GPP); Severity Indices of Personality Problems (SIPP-118) [16], the Big Five personality traits, Adaptive Test of Personality Disorder [18], Personality Inventory for DSM-5 (PID-5) [18, 19, 20]. Studies have shown high internal consistency of the LPFS-SR, which, according to Morey [16], shows high subcomponent intercorrelation and supports the idea of the LPFS-SR as a single dimension that can manifest itself both interpersonally and intrapersonally.

Despite its international significance, research on the clinical utility of dimensional diagnostic tools within the Czech population remains relatively limited. Most recently, Heissler* et al*. [21] conducted a psychometric evaluation of the Semi-structured Interview for Personality Functioning (STiP-5.1), while Riegel* et al*. [22] assessed the reliability and validity of the Personality Inventory for DSM-5-Brief Form Plus Modified (PID5BF+M) on two non-clinical samples of a total of 1654 Czech volunteers, and Doubkova* et al*. [23] investigated the psychometric properties and clinical utility of the Level of Personality Functioning Scale-Brief Form (LPFS-BF 2.0) in relation to the STiP-5.1. In all cases, these instruments showed satisfactory psychometric properties.

While a relatively broad range of studies has focused on the application of the LPFS-SR in non-clinical and mixed psychiatric samples, the evidence of data from patients with SUD is still rather limited. To our knowledge, only two studies have thus far investigated self-reported LPFS in this context. Bach and Hutsebaut [24] explored the utility of the LPFS-Brief Form 2.0 among incarcerated male individuals with substance use disorder and individuals receiving mental health care in an outpatient setting, while Papamalis* et al*. [4] examined the treatment engagement of service users at an inpatient unit based personality functioning, utilizing the Severity Indices of Personality Problems (SIPP-118). The goal of this study was to examine self-reported personality functioning among Czech patients with SUD, in order to contribute to the growing body of research on this vulnerable population, where more precise personality assessment might have a significant role in treatment planning and the course of substance use disorder treatment. To fulfill this objective, the evaluation of the psychometric properties of the Czech version of the LPFS-SR was a necessary first step. In line with previous studies utilizing the LPFS-SR [16, 18, 19, 25, 26], we anticipated satisfactory values of internal consistency, confirmation of the proposed one-factor structure, high correlations with personality traits as measured by the PID-5, and the instrument’s ability to discriminate according to the clinical status of the respondents. Moreover, we expected individuals with SUD to score higher on the LPFS-SR than those from the general population. Regarding gender differences, studies by Le Corff *et al*. [27] and Wu* et al*. [28] suggested that personality functioning remains consistent across genders. However, Wu* et al*. [28] reported that the LPFS-BF exhibited additional variance in predicting borderline personality features. Given the frequent co-occurrence of SUD and personality disorders, as well as research indicating a higher prevalence of borderline personality disorder among females [29], we aimed to explore potential gender-related differences by calculating gender-specific cut-off scores. This approach could further enhance the practical utility of the LPFS-SR in clinical settings.

## 2. Methods 

### 2.1 Samples and Procedures

Two research samples were used in this study. Sample 1 consisted of individuals diagnosed with substance use disorder (SUD), while Sample 2 consisted of volunteers from the general population. All participants took part voluntarily, anonymously and without financial compensation. Informed consent was obtained from all participants, who were also given the opportunity to withdraw from the study at any time without providing a reason. The informed consent process and study protocol were approved by the Ethics Committee of the General University Hospital in Prague (Approval No. 126/21 S-IV). Following the exclusion of incomplete demographic or questionnaire data, a total of 865 participants were included in the study. Eligibility criteria for both samples required participants to be over 18 years of age and fluent readers of the Czech language. For the clinical sample, the additional inclusion criterion was a diagnosis of SUD, while exclusion criteria included the presence of other addictions (unless co-occurring with SUD), acute psychotic symptoms, current intoxication, or organic brain disorders associated with significant cognitive impairment.

Sample 1 consisted of 368 participants (42.54%) diagnosed with SUD, recruited from seven specialized SUD treatment facilities across Czechia. To ensure broad representation, recruitment included institutions providing outpatient and inpatient hospital care, as well as therapeutic communities. However, lower-threshold services, such as harm reduction interventions and units dispensing opioid agonist therapy, were excluded due to potential risk of data distortion caused by acute intoxication or low participant engagement. The SUD diagnosis was determined using a standard clinical interview based on International Classification of Diseases 10th Revision (ICD-10)/DSM-IV criteria, conducted by experienced psychiatrists. Additional information regarding participants’ histories of substance use and gambling behavior was obtained from the demographic questionnaire. The gender distribution within Sample 1 comprised 252 males (68.48%) and 116 females (31.52%), with an age range of 18–73 years and a mean age of 39.44 ± 11.19 years. Based on the data obtained from this questionnaire, the distribution of substance use and behavioral addiction within the sample was as follows: alcohol use disorder (n = 27, 73.64%); prescription medication misuse (n = 123, 33.42%); illicit drug use disorder (n = 206, 55.98%); gambling disorder (n = 61, 16.58%).

Sample 2 consisted of 497 participants (57.46%), including working professionals, university students from various fields of study, and retirees. The gender distribution within this sample was 171 males (34.41%) and 326 females (65.59%), with an age range of 18–75 years and a mean age of 33.47 ± 12.13. Participants were recruited through convenience sampling. The representation of substance use and gambling behavior in Sample 2 was as follows: alcohol use disorder (n = 20, 4.02%); prescription medication misuse (n = 16, 3.22%); illicit drug use disorder (n = 19, 3.82%); gambling behavior (n = 2, 0.40%).

Data collection was conducted using a paper-and-pencil method. Trained administrators were present during the whole administration process to help if needed. For Sample 1, prior to data collection, administrators coordinated with the heads of psychiatric institutions and departments to determine the appropriate time and location for administration. Participants required only a pen to complete the assessment. The time required to complete the test battery ranged from 40 to 90 minutes.

### 2.2 Measures

#### 2.2.1 Level of Personality Functioning Scale-Self Report (LPFS-SR) 

The LPFS-SR is an 80-item self-report scale for adults, with each item rated on a four-point Likert scale ranging from 1 (Totally False, Not at All True) to 4 (Very True). The scoring system is based on multiplying the scale value of the item by its corresponding weight value. Each of the 80 items belongs to one of four subdomains, representing the core functions of personality toward the self (Identity, Self-Direction) and others (Empathy, and Intimacy). The total score reflects the severity of impairment in general personality functioning. The translation of LPFS-SR from English into Czech language followed a meticulous procedure. Initially, two team members independently translated each item into Czech. The preliminary Czech version, developed through a consensus between the translators, was then forwarded to a third translator with native-level proficiency in English for back-translation. This back-translated version was subsequently review by the original author of the LPFS-SR to ensure the accuracy and fidelity of the translation. The Czech version of the LPFS-SR is available for free at: LPFS-SR-CZ.pdf (hogrefe.cz).

#### 2.2.2 Personality Inventory for DSM-5 (PID-5)

The PID-5 is a 220-item self-report inventory, originally created to correspond to Criterion B of the AMPD. Each item is evaluated on a four-point Likert scale, ranging from 0 (Very False or Often False) to 3 (Very True or Often True). It assesses 25 trait facets in five broad domains, each primarily defined by three key facets: Negative Affectivity (emotional lability, anxiousness, separation insecurity); Detachment (withdrawal, anhedonia, intimacy avoidance); Antagonism (manipulativeness, deceitfulness, grandiosity); Disinhibition (irresponsibility, impulsivity, distractibility); Psychoticism (unusual beliefs and experiences, eccentricity, perceptual dysregulation) [30]. In this study, the Cronbach’s alphas coefficients ranged from 0.52 (Sample 1, suspiciousness) to 0.94 (Sample 2, eccentricity). The PID-5 was used as an external measure to assess the criterion validity of the LPFS-SR.

#### 2.2.3 Sociodemographic Questionnaire

Additionally, essential demographic information, including age, gender, educational background, and type of addiction, was collected through a questionnaire specifically designed for this purpose.

### 2.3 Statistical Analysis

To assess the internal consistency of the LPFS-SR, Cronbach’s alpha coefficients were calculated for each subdomain and the total score. Principal component analysis (PCA) was used to identify the strong underlying factor structure of the LPFS-SR components. Gender differences were examined using a two-way analysis of variance (ANOVA), with gender and diagnosis as the two categorical variables. Logistic regression was performed to determine cut-off values between groups. Prior to this analysis, each facet was assessed individually using the interquartile range (IQR) criterion to detect and exclude outliers, preventing them from influencing cut-off. To enhance the stability of the results, a five-fold cross-validation was implemented. Five logistic regression models were developed using five training samples, and the mean area under the curve (AUC) was calculated based on the test samples. A 95% confidence interval (CI) for the AUC was calculated using 2000 stratified bootstrap replicates. The optimal threshold probability was identified as the point where the average number of false-negative and false-positive patients across the five models was minimized. To balance the ratio of patients and non-patients, weights (50% for patients and 50% for non-patients) were assigned during minimization. The resulting cut-off values were obtained from logistic regression applied to the entire dataset of the group. To assess the validity of the LPFS-SR, Pearson’s correlation coefficient was calculated with the PID-5 domains and facets. All statistical analyses were performed using the R 4.2.2 statistical software (R Foundation for Statistical Computing, Vienna, Austria) (https://www.R-project.org/).

## 3. Results

The internal consistency of the LPFS-SR, as estimated by Cronbach’s alpha, yields values ≥0.66 for each subdomain and the total score. The Cronbach’s alpha values are shown in Table 1. The inter-scale correlation varies from 0.74 to 0.94, indicating a sufficient level of reliability. A significant effect of gender was observed only for the Identity subdomain (*p* = 0.04), while no significant differences were found for Self-Direction (*p* = 0.64), Empathy (*p* = 0.55), Intimacy (*p* = 0.51), or the Total score (*p* = 0.58).

**Table 1.** **Cronbach’s alphas for both samples**.

	Sample 2 (Control)		Sample 1 (Clinical)	
	Cronbach’s alpha	M (SD)	Cronbach’s alpha	M (SD)
Identity	0.86	72.18 (19.61)	0.86	94.25 (23.99)
Self-Direction	0.86	49.20 (14.18)	0.86	66.69 (19.31)
Empathy	0.69	36.78 (9.75)	0.66	48.46 (12.81)
Intimacy	0.83	57.35 (15.43)	0.82	75.30 (20.62)
Total score	0.95	215.51 (52.69)	0.94	284.70 (68.09)

M, mean score; SD, standard deviation. Control, control group; Clinical, clinical group.

In Sample 1, PCA of the four LPFS-SR subscales revealed a single component accounting for 78.21% of the variance. In Sample 2, the variance explained was 79.20%. The PCA loadings for both samples are shown in Table 2.

**Table 2.** **Principal component analysis (PCA) loadings for both samples**.

	Sample 2 (Control)		Sample 1 (Clinical)	
	Component 1	Component 2	Component 1	Component 2
Identity	0.51	0.49	0.51	0.38
Self-Direction	0.51	0.50	0.50	0.58
Empathy	0.50	–0.50	0.50	–0.39
Intimacy	0.50	–0.51	0.49	–0.60

In Tables 3,4 the mean LPFS-SR scores for males and females are provided separately, further divided by sample groups; the number of observations for the scores differs because of the individual elimination of outliers. The average AUC in both groups reached high values ranging from 0.73 (female Intimacy) to 0.80 (male total score). Notably, all the cut-off values, except for Intimacy, are higher for the female group, a finding consistent with the observed mean scores in each group.

**Table 3.** **Cut-off and mean scores for males, logistic regression coefficients**.

	n	Clinical	M (SD)	M (SD)	Intercept (*p*-value)	Coefficient (*p*-value)	AUC (CI)	Cut-off
			(Control)	(Clinical)				
Identity	422	251	69.82 (19.41)	92.98 (22.15)	–0.346 (<0.001)	0.008 (<0.001)	0.79	80.84
							(0.74, 0.83)	
Self- Direction	423	252	49.27 (15.29)	66.09 (18.41)	–0.371 (<0.001)	0.012 (<0.001)	0.77	48.06
							(0.72, 0.81)	
Empathy	421	251	36.77 (9.51)	48.39 (12.41)	–0.464 (<0.001)	0.019 (<0.001)	0.77	39.65
							(0.72, 0.81)	
Intimacy	418	249	56.40 (14.75)	75.40 (20.01)	–0.319 (<0.001)	0.009 (<0.001)	0.78	70.58
							(0.73, 0.82)	
Total score	422	251	213.11 (54.54)	283.08 (64.96)	–0.493 (<0.001)	0.003 (<0.001)	0.80	229.44
							(0.76, 0.84)	

n, Number of participants; AUC, Area under the curve; CI, Confidence interval.

**Table 4.** **Cut-off and mean scores for females, logistic regression coefficients**.

	n	Clinical	M (SD)	M (SD)	Intercept	Coefficient (*p*-value)	AUC (CI)	Cut-off
			(Control)	(Clinical)	(*p*-value)			
Identity	440	114	73.42 (19.62)	95.50 (26.22)	–0.221 (0.004)	0.010 (<0.001)	0.75	83.79
							(0.69, 0.80)	
Self- Direction	433	110	48.74 (12.91)	65.94 (19.61)	–0.065 (0.354)	0.011 (<0.001)	0.76	67.57
							(0.70, 0.81)	
Empathy	433	114	35.97 (8.51)	47.82 (12.85)	–0.166 (0.031)	0.017 (<0.001)	0.77	42.22
							(0.71, 0.82)	
Intimacy	438	113	57.38 (15.10)	72.58 (19.40)	–0.157 (0.036)	0.011 (<0.001)	0.73	59.55
							(0.67, 0.78)	
Total score	437	113	215.79 (50.38)	282.99 (70.00)	–0.286 (<0.001)	0.003 (<0.001)	0.78	248.87
							(0.72, 0.83)	

The correlations between the LPFS-SR and PID-5 are shown in Table 5. They varied from 0.36 to 0.76 for the LPFS-SR and PID-5 domains, and from 0.21 to 0.77 for the LPFS-SR and PID-5 facets, indicating sufficient criterion validity of the LPFS-SR.

**Table 5.** **Pearson’s correlation coefficients of LPFS-SR and PID-5 with *p*-values in both samples**.

Facets	Identity	Self- Direction	Empathy	Intimacy	Total score	Cronbach’s alpha	Cronbach’s alpha	Cronbach’s alpha
							(Control)	(Clinical)
Anhedonia	0.61 (<0.001)	0.68 (<0.001)	0.55 (<0.001)	0.59 (<0.001)	0.67 (<0.001)	0.83	0.84	0.81
Anxiousness	0.70 (<0.001)	0.63 (<0.001)	0.50 (<0.001)	0.55 (<0.001)	0.67 (<0.001)	0.89	0.91	0.87
Attention Seeking	0.31 (<0.001)	0.23 (<0.001)	0.27 (<0.001)	0.23 (<0.001)	0.29 (<0.001)	0.89	0.91	0.90
Callousness	0.44 (<0.001)	0.45 (<0.001)	0.52 (<0.001)	0.53 (<0.001)	0.52 (<0.001)	0.84	0.84	0.84
Deceitfulness	0.46 (<0.001)	0.44 (<0.001)	0.46 (<0.001)	0.44 (<0.001)	0.49 (<0.001)	0.84	0.85	0.85
Depressivity	0.74 (<0.001)	0.75 (<0.001)	0.63 (<0.001)	0.66 (<0.001)	0.77 (<0.001)	0.93	0.93	0.92
Distractibility	0.71 (<0.001)	0.73 (<0.001)	0.60 (<0.001)	0.57 (<0.001)	0.73 (<0.001)	0.89	0.90	0.87
Eccentricity	0.66 (<0.001)	0.58 (<0.001)	0.61 (<0.001)	0.57 (<0.001)	0.66 (<0.001)	0.94	0.94	0.93
Emotional Lability	0.68 (<0.001)	0.56 (<0.001)	0.49 (<0.001)	0.54 (<0.001)	0.63 (<0.001)	0.85	0.87	0.83
Grandiosity	0.32 (<0.001)	0.27 (<0.001)	0.40 (<0.001)	0.38 (<0.001)	0.37 (<0.001)	0.80	0.81	0.80
Hostility	0.59 (<0.001)	0.50 (<0.001)	0.54 (<0.001)	0.54 (<0.001)	0.60 (<0.001)	0.84	0.84	0.83
Impulsivity	0.56 (<0.001)	0.52 (<0.001)	0.48 (<0.001)	0.48 (<0.001)	0.56 (<0.001)	0.74	0.71	0.70
Intimacy Avoidance	0.31 (<0.001)	0.37 (<0.001)	0.37 (<0.001)	0.43 (<0.001)	0.40 (<0.001)	0.76	0.78	0.75
Irresponsibility	0.61 (<0.001)	0.67 (<0.001)	0.55 (<0.001)	0.57 (<0.001)	0.66 (<0.001)	0.82	0.80	0.79
Manipulativeness	0.31 (<0.001)	0.26 (<0.001)	0.33 (<0.001)	0.31 (<0.001)	0.33 (<0.001)	0.80	0.81	0.79
Perceptual Dysregulation	0.68 (<0.001)	0.65 (<0.001)	0.63 (<0.001)	0.61 (<0.001)	0.70 (<0.001)	0.85	0.87	0.82
Perseveration	0.68 (<0.001)	0.62 (<0.001)	0.57 (<0.001)	0.58 (<0.001)	0.68 (<0.001)	0.81	0.84	0.73
Restricted Affectivity	0.27 (<0.001)	0.30 (<0.001)	0.37 (<0.001)	0.35 (<0.001)	0.34 (<0.001)	0.66	0.70	0.61
Rigid Perfectionism	0.37 (<0.001)	0.21 (<0.001)	0.33 (<0.001)	0.35 (<0.001)	0.35 (<0.001)	0.87	0.89	0.84
Risk Taking	0.27 (<0.001)	0.25 (<0.001)	0.25 (<0.001)	0.24 (<0.001)	0.28 (<0.001)	0.88	0.87	0.89
Separation Insecurity	0.54 (<0.001)	0.46 (<0.001)	0.45 (<0.001)	0.48 (<0.001)	0.54 (<0.001)	0.82	0.83	0.81
Submissiveness	0.46 (<0.001)	0.42 (<0.001)	0.29 (<0.001)	0.29 (<0.001)	0.41 (<0.001)	0.74	0.75	0.74
Suspiciousness	0.60 (<0.001)	0.55 (<0.001)	0.60 (<0.001)	0.61 (<0.001)	0.64 (<0.001)	0.58	0.57	0.52
Unusual Beliefs & Experiences	0.51 (<0.001)	0.43 (<0.001)	0.53 (<0.001)	0.47 (<0.001)	0.53 (<0.001)	0.83	0.83	0.82
Withdrawal	0.45 (<0.001)	0.45 (<0.001)	0.50 (<0.001)	0.56 (<0.001)	0.53 (<0.001)	0.86	0.87	0.82
Domains								
Negative Affect	0.76 (<0.001)	0.65 (<0.001)	0.56 (<0.001)	0.61 (<0.001)	0.72 (<0.001)			
Detachments	0.55 (<0.001)	0.60 (<0.001)	0.57 (<0.001)	0.63 (<0.001)	0.64 (<0.001)			
Antagonism	0.41 (<0.001)	0.36 (<0.001)	0.45 (<0.001)	0.43 (<0.001)	0.45 (<0.001)			
Disinhibition	0.74 (<0.001)	0.75 (<0.001)	0.64 (<0.001)	0.63 (<0.001)	0.76 (<0.001)			
Psychoticism	0.70 (<0.001)	0.62 (<0.001)	0.67 (<0.001)	0.62 (<0.001)	0.72 (<0.001)			

## 4. Discussion

This research aimed to explore personality functioning in individuals with substance use disorder (SUD). To contribute to the harmonization of cross-cultural personality disorder diagnostics, we translated the LPFS-SR into Czech and evaluated its validity and reliability across two clinically distinct samples. As expected, we confirmed satisfactory values of internal consistency, the proposed one-factor structure, as well as the criterion validity of the instrument. Moreover, the gender-specific cut-off scores offer further confirmation of the discriminating capacity of the LPFS-SR in distinguishing based on the clinical status of the respondents, expanding its applicability in routine clinical practice. The implications of these findings are discussed in detail below.

SUD is often linked to personality pathology, profoundly affecting multiple aspects of an individual’s life and, consequently, personality functioning. Thus, the observed differences between the clinical and general populations were consistent with expectations. In line with the main goal of this study, we determined gender-specific cut-off scores for distinguishing between the personality functioning of individuals with SUD from those of the general population. Across all dimensions measured, including total personality functioning, males consistently scored lower than females, with the exception of the Intimacy dimension. Gender differences were insignificant, except for Identity, where females scored significantly higher. This pattern suggests that females may exhibit greater impairments in personality functioning when assessed comprehensively. These findings align with previous research highlighting gender differences in the prevalence and manifestation of personality pathology. For instance, Bradley* et al*. [29] reported that females are more frequently diagnosed with borderline personality disorder (BPD), which may reflect more severe manifestations of emotional lability/negative affectivity in females compared to males. Within the AMPD framework, this assumption was recently further supported in research by Riegel* et al*. [30], where Identity dimension of LPFS-SR and negative affectivity demonstrated significant correlation.

The cut-off total scores for the LPFS-SR were found to be 229.44 for males and 248.87 for females, which is lower than the cut-off of 347.1 reported in the original version of the LPFS-SR [31]. The Persian version of the LPFS-SR [25] determined 306.11 as the total score cut-off, while the Portuguese version [26] identified an optimal total cut-off of 272.0. The variability of results can likely reflect the differences in sample sizes and the characteristics of the categories that were compared. For example, Pires* et al*. [26] compared 40 participants diagnosed with PD to a community sample, while Hemmati* et al*. [25] compared 142 participants with PD to college students. In contrast, Morey [31] involved 306 non-clinical participants, whereas our study compared 368 patients with SUD to 497 community volunteers. The lower total personality functioning scores observed in individuals with SUD, relative to outpatients in mental health treatment captured in Bach & Hutsebaut [24] suggests that individuals with SUD may exhibit less severe overall personality pathology compared to outpatients in mental health treatment. These findings underscore the need for further research to explore population-specific distinctions in personality pathology. Additionally, cross-cultural differences might contribute to the variability observed in total cut-off scores across the studies. For instance, variations in the English, Portuguese, Persian, Czech, and Polish versions of the LPFS-SR could influence the determination of cut-off scores, emphasizing the necessity of further research for a more comprehensive understanding of these cultural differences.

The Czech LPFS-SR demonstrated high internal consistency across both the samples, with Cronbach’s alpha values aligning closely with those reported in the original validation studies with the LPFS-SR [16, 18] and comparable to findings from cross-cultural adaptations [25, 26]. The calculated proportion variance among the four LPFS-SR subscales providing a single component is in line with the original research by Morey [16]. It closely resembles the ICD-11 dimensional approach to PDs, as the ICD-11 framework also conceptualizes impairment in both the self and the interpersonal functioning of an individual as an essential condition when considering the severity of personality psychopathology. Notably, self-other impairment is recognized as a fundamental concept in dimensional diagnostic tools, indicating a high degree of compatibility between measures originally developed for the AMPD, ICD-11, or PD model rooted in object relations theory [32].

Regarding the discriminating capacity of the Czech LPFS-SR to distinguish between patients with SUD and the general population, the Czech LPFS-SR exhibits considerable accuracy, showing high sensitivity and specificity at the determined cut-off. This finding confirms conclusions about the good discriminating capacity of the LPFS-SR reported earlier by Pires* et al*. [26] and Hemmati* et al*. [25].

From the perspective of the criterion validity of the LPFS-SR, Hopwood* et al*. [18] demonstrated that maladaptive personality traits generally correlate with overall personality pathology. Accordingly, we incorporated the PID-5 in the validation process of the LPFS-SR. Our data indicate a robust overall correlation with the PID-5 domains, suggesting sufficient concurrent criterion validity. However, Hopwood* et al*. [18] found that certain types of maladaptive personality traits do not exhibit a strong association with general personality pathology. Specifically, their findings indicate a weak association with every component of the LPFS-SR and risk-taking. Similarly, in our research, certain PID-5 facets exhibited low correlation with the total LPFS-SR score and its subdomains. Notably, we found low correlation between the risk-taking and attention-seeking facets and all LPFS-SR components. At the PID-5 domain level, Antagonism showed the lowest correlation. All other facet correlations attained values exceeding 0.50. In Roche* et al*. [20], Antagonism also demonstrated the weakest association with the LPFS-SR, along with Disinhibition. When assessing the criterion validity of the Polish version of the LPFS-SR, Łakuta* et al*. [19] also found a low correlation with the LPFS-SR and Disinhibition. A possible explanation is that Antagonism is a facet that encompasses domains such as Manipulativeness, Deceitfulness, and Grandiosity. Individuals exhibiting these traits may want to present themselves as more socially acceptable. Although we sought to mitigate these inclinations by making the questionnaires anonymous, the possibility of social desirability bias influencing their responses cannot be entirely ruled out.

Overall, our findings regarding the reliability, validity, and discriminating capacity of the Czech version of the LPFS-SR are in accordance with previous research [16, 18, 19, 20]. Nevertheless, several limitations in the context of this study must be acknowledged. One concern is the gender ratio imbalance observed within both samples that could raise some concerns about the generalizability of the findings across both populations. To address this, we calculated the cut-off scores for males and females separately in both samples. Another potential limitation refers to the methodological aspects of data collection. The extensive length of the questionnaires may pose a challenge for participants, potentially leading them to question skipping, loss of focus, or exchange of answers. This issue, commonly referred to as non-response bias, could have occurred in some cases, which, according to Sedgwick [33], is the most common limitation of questionnaire surveys. While data cleaning procedures were applied, it remains possible that modifying the questionnaire order or administering it in shorter segments could have a positive effect on their response quality, especially regarding the number of missing items in some scales. From this perspective, supplementing the self-assessment with tools such as the STiP-5.1 could significantly enhance the reliability of respondents’ statements. However, due to time constraints and the aim of obtaining the largest possible number of respondents from institutions throughout the country, conducting structured interviews was not feasible. The use of convenience sampling was employed in this study, which carries its own limitations. While convenience samples do not necessarily represent broader populations, they can offer high internal validity if the research design is rigorous and the data analysis is sound. However, such samples often limit external validity, which makes the process of generalization challenging. Generalization from convenience samples is only viable if the sample is randomly selected from the target population [34]. Although this study is still rather exploratory in its nature, future research should consider quota sampling for more precise normative data. Another limitation was the lack of exploration of comorbid psychiatric disorders, such as anxiety or depression, which are highly prevalent in individuals with SUD. These comorbidities could influence personality functioning scores, that is why future research should consider the assessment of these co-occurring disorders. Finally, it is necessary to note that our study focused primarily on individuals with SUD that meet high-threshold criteria. This decision was intentional, considering the elevated risk of acute intoxication and higher dropout rates within the low-threshold individuals. Nevertheless, it is obvious from clinical practice that low-threshold clients exhibit a substantial degree of personality pathology. Including this population in future research could broaden the scope of our findings.

## 5. Conclusion

We demonstrated that the LPFS-SR is a reliable and valid tool for assessing personality functioning in Czech individuals with SUD. The population-specific cut-off scores provided in this study can enhance the practical applicability of the tool in routine clinical practice. Understanding the role of personality functioning in the treatment process of individuals with SUD can help identify specific vulnerabilities and needs stemming from the depth of impairment in relation to the self and others. Addressing these factors may contribute to reducing the risk of premature treatment termination or possible relapses. We believe that population-specific diagnostic thresholds facilitate the interpretation of scores in treatment planning and enable more meaningful monitoring of treatment effectiveness.

## Availability of Data and Materials

The data from this study are available upon request.
